# *Tetraselmis jejuensis* sp. nov. (Chlorodendrophyceae), a Euryhaline Microalga Found in Supralittoral Tide Pools at Jeju Island, Korea

**DOI:** 10.3390/plants10071289

**Published:** 2021-06-24

**Authors:** Jun-Ho Hyung, Eun-Joo Kim, Seung-Joo Moon, Nam Seon Kang, Jaeyeon Park

**Affiliations:** 1 Environment and Resource Convergence Center, Advanced Institute of Convergence Technology, Suwon 16229, Korea; hjh1120@snu.ac.kr (J.-H.H.); kej1005@snu.ac.kr (E.-J.K.); sjmoon04@snu.ac.kr (S.-J.M.); 2 Department of Taxonomy and Systematics, National Marine Biodiversity Institute of Korea, Seocheon 33662, Korea

**Keywords:** microalgae, *Tetraselmis* spp., morphology, ultrastructure, salinity

## Abstract

We found the euryhaline microalga, *Tetraselmis jejuensis* sp. nov., which was adapted to supralittoral tide pools with salinities varying from 0.3–3.1%. Fifteen strains of *T*. *jejuensis* were isolated from Daejeong (DJ) and Yongduam (YO), and clonal cultures were established in the laboratory. Morphological characterization revealed that the cells have a compressed shape, four flagella emerging from a depression near the apex in two opposite pairs, a cup-shaped chloroplast containing one pyrenoid surrounded by starch, and eyespot regions not located near the flagellar base. *T*. *jejuensis* cells showed distinct characteristics compared to other *Tetraselmis* species. First, a regular subunit pattern with honeycomb-like structures was predominantly displayed on the surface in the middle of the cell body. Second, the pyrenoid was invaded by both cytoplasmic channels comprising electron-dense material separated from the cytoplasm, and two branches of small cytoplasmic channels (canaliculi) in various directions, which characterize the subgenus *Tetrathele*. Eyespot regions containing a large number of osmiophilic globules, packed closely together and arranged in subcircular close packing of diverse sizes, were dispersed throughout the chloroplast. In the phylogenetic analysis of small subunit (SSU) rDNA sequences, the 15 strains isolated from DJ and YO separated a newly branched clade in the Chlorodendrophyceae at the base of a clade comprising the *T. carteriiformi/subcordiformis* clade, *T. chuii*/*suecica* clade, and *T. striata/convolutae* clade. The strains in the diverging clade were considered to belong to the same species. The SSU rDNA sequences of the DJ and YO strains showed a maximum difference of 1.53% and 1.19% compared to *Tetraselmis suecica* (MK541745), the closest species of the family based on the phylogenetic analysis, respectively. Based on morphological, molecular, and physiological features, we suggest a new species in the genus *Tetraselmis* named *Tetraselmis jejuensis*, with the species name “jejuensis” referring to the collection site, Jeju Island, Korea.

## 1. Introduction

Marine microalgae have been recognized as promising resources for food supplements, cosmeceuticals, and biofuels [1,2,3]. For commercial applications, the establishment of mass cultivation systems is necessary for the selected monoculture microalgae. Open pond culture systems have been successfully used for mass cultivation because outdoor algal culture reduces the cost of culture maintenance and is easily controlled compared to indoor tanks or photobioreactors that require a large area, heating balance, and sterilization [4,5,6,7]. However, long-term cultures of microalgae in these systems are limited to a few species as they are exposed to extreme environments, such as varying salinity and nutrients due to evaporation or rainfall [8,9,10]. Thus, it is necessary to explore suitable microalgal species for successful monoclonal culture and under changing environmental conditions.

Euryhaline marine microalgae, of the genus *Tetraselmis*, have been recently studied for efficient biofuel production [10,11,12,13]. They survive and maintain their robustness in outdoor culture conditions over a long period by controlling cytoplasmic ion homeostasis under severe osmotic stress by regulating ion concentrations [7,10,14]. The genus *Tetraselmis* is a member of Chlorodendrophyceae, a small class of green algal lineages that comprises marine and freshwater scaly quadriflagellates [15]. Traditionally classified within Prasinophytes, Chlorodendrophyceae are currently nested within the core Chlorophyta, including the Ulvophyceae, Trebouxiophyceae, and Chlorophyceae [16,17,18]. Phylogenetic analysis based on 18S rDNA sequences indicates that Chlorodendrophyeceae belong to the core Chlorophyta as a deep diverging lineage [15,19,20,21]. As one of the key characteristics of the core Chlorophyta, cell division of *Tetraselmis* species is mediated by a phycoplast, including closed mitosis [22,23]. The morphological features of genus *Tetraselmis* are characterized by a compressed shape, four flagella of equal length emerging from a slit at the apical base, a single large chloroplast containing one pyrenoid and a conspicuous eyespot, and a nucleus that is parallel to the pyrenoid and flagellar base. *Tetraselmis* species are commonly found in estuaries, tide pools, and brackish ponds, where environmental fluctuations often occur because of rain storms, wave action, and evaporation [24,25].

The coastlines of Jeju Island are composed of basalt-based rocky shores. In supralittoral tide pools located above the high-tide line, several depressions formed by the vesicular textures of basalt are occasionally submerged, either under seawater from wave splash, winds, and storms, or under freshwater from heavy rainfall [26]. Microorganisms adapted to these habitats show high tolerance to extreme changes in salinity, temperature, and nutrient composition [27]. Although *Tetraselmis gracilis* isolated from the seawater of Jeju coast has been reported previously by [28], *Tetraselmis* species living in the supralittoral zone on Jeju Island have been much less explored. Moreover, traditional species circumscriptions can lead to species misidentification because diagnosis for cellular characterization is very complex, even though the morphological and ultrastructural characteristics of many *Tetraselmis* species have been well studied by light microscopic observations [29,30,31]. To prevent the commercial use of incorrect species, which could lead to economic losses, it is important to establish correct species assignments. Recently, ultrastructural investigations of species using electron microscopy have provided more distinctive features to allow species delimitation [22,32,33,34,35,36]. Phylogenetic analysis of species using DNA sequences has also presented useful taxonomic criteria to understand intraspecific variation and to identify similar species [37].

For collecting robust microalgae species, we investigated diverse sites with various environmental conditions, and about 50 strains of microalgae were collected. Among them, we first found a species of *Tetraselmis* adapted to the supralittoral tide pools on Jeju Island, and successfully established clonal cultures in the laboratory. Its morphological details and ultrastructure were investigated using light microscopy, scanning electron microscopy, and transmission electron microscopy. Molecular taxonomic identification of cells was performed by phylogenetic analysis based on the SSU rDNA sequences. 

## 2. Results

### 2.1. Morphological Analysis of Tetraselmis jejuensis

**Figure 1:**
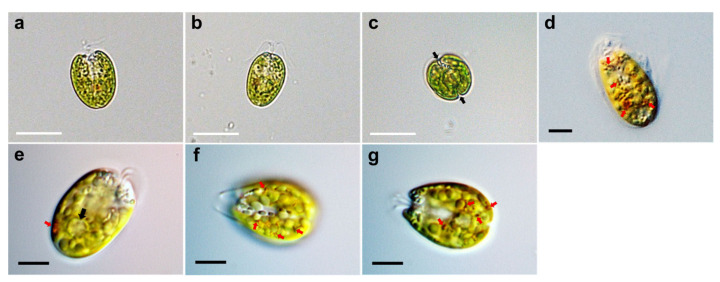
Micrographs of *Tetraselmis jejuensis* taken using light microscopy. (**a**) A broad lateral view, (**b**) a narrow lateral view, and (**c**) an antapical view of the cells. Two distinct creases are observed in the antapical view (black arrows). (**d**–**g**) Light microscopic images obtained using differential interference contrast (DIC). The scaly green flagellates showed a compressed shape, flagella attached to the bottom of the apical depression, a yellow-green colored chloroplast, pyrenoid matrix at the posterior part (black arrow), and conspicuous eyespot regions containing orange-red pigment granules (red arrows), spread out in the chloroplast. Scale bars (white) = 20 µm. Scale bars (black) = 5 µm.

Under light microscopy, the cells showed a compressed shape, flagella emerging from a depression at the apical base, a cup-shaped chloroplast which was yellow-green in color, and distinct creases that were parallel in the middle of the cell body (Figure 1a–c). The ovoid cells showed either a broad or narrow face in light micrographs. The anteroposterior (AP) length and width of the cells in the broad lateral view was 13.0–20.8 µm (16.7 ± 2.5 µm), and 6.5–16.3 µm (11.3 ± 2.7 µm), respectively (n = 40). The width from the narrow face corresponding to the depth in the broad view was 9.8–13.0 µm (11.7 ± 0.8 µm, n = 30) and was shorter than that in the broad lateral views. Light microscopic images using differential interference contrast (DIC) showed a scaly theca covering the cell body, a pyrenoid matrix located at the posterior part, and numerous orange-red eyespot regions dispersed throughout the chloroplast (Figure 1d–g).

**Figure 2:**
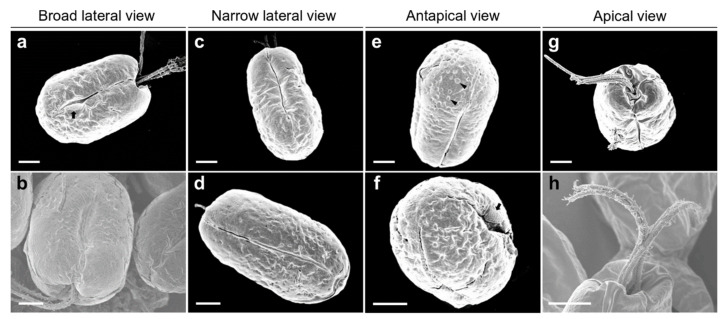
Micrographs of *Tetraselmis jejuensis* obtained using scanning electron microscopy. (**a**,**b**) Broad lateral views of cells showing depressions on the apical side. Distinct creases in the middle, and a regular subunit pattern (black arrow) on the cell surface adjacent to the middle furrow were observed. (**c**,**d**) Narrow lateral views of cells containing deep folded lines in the middle. Depressions were obscured from the apical peaks. (**e**,**f**) Antapical views of cells showing granular scales on the surface (black arrowheads). A regular subunit pattern was exhibited on the surface near the furrow (black arrow). (**g**,**h**) Apical views of the cells showing four flagellar hairs in 2 opposite pairs. Scale bars = 2 µm.

**Figure 3:**
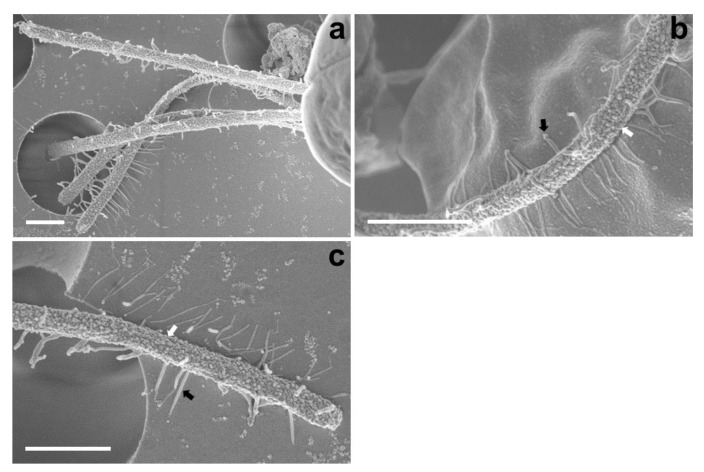
Micrographs of the flagella in *Tetraselmis jejuensis* obtained using scanning electron microscopy. (**a**) Four flagella were observed at the bottom of the cell body. (**b**,**c**) Structure of the cell flagella; microtubules showing thick, rod-shaped, and blunt-ended stalks (white arrows) and thin pit hairs (black arrows). Scale bars = 1 µm.

**Figure 4:**
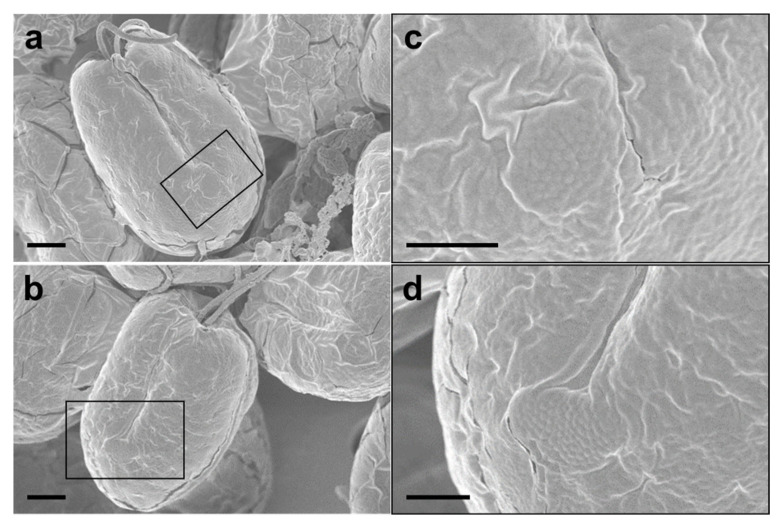
Regular subunit patterns and honeycomb-like structures observed in SEM micrographs of *Tetraselmis jejuensis*. (**a**,**b**) The unique patterns are observed in the broad lateral views. (**c**,**d**) Magnified views of regions highlighted by black squares in the adjacent panels. Scale bars = 2 µm for (**a**), (**b**). Scale bars = 1 µm for (**c**,**d**).

The SEM micrographs of *Tetraselmis jejuensis* in the broad lateral view showed a slightly compressed shape, with four flagella emerging from a slit at the base of the apical depression, and distinct creases in the middle of the cell body (Figure 2a,b). The width of the apical depression was approximately 0.7–1.3 µm (mean ± standard deviation = 1.0 ± 0.14 µm, n = 20). A deep-folded line in the middle of the cell body extended longitudinally from the apical depression to the posterior part of the cell. The linear crease was also observed in the middle when viewed from the narrow lateral side of the cells, but the apical depression was obscured from this view (Figure 2c,d). In the antapical view of the cells, some granular scales were spread over the cell surface, especially at the antapical side, and distinct creases in the middle were observed from each broad or narrow lateral side (Figure 2e,f). One crease from the lateral side was parallel to the other from the opposite side. As a common feature of motile cells of *Tetraselmis* species, the four flagella of the cells were arranged in two opposite pairs at the apical positions (Figure 2g,h and Figure 3a). In the magnified views of the flagella in the cells using SEM, the structure of the flagellar filaments was composed of thick, blunt-ended, and rod-shaped microtubules suitable for dynamic bending motions, and showed a fairly large number of thin pit hairs measuring approximately 0.2–0.8 µm (0.5 ± 0.13 µm, n = 30) in length, attached to the stalks (Figure 3b,c). Notably, most cells of *Tetraselmis jejuensis* contained regular subunit patterns on the cell surface near the posterior part adjacent to the middle furrow on the broad lateral side Figure 2a,f and Figure 4a,b). The magnified views of the SEM micrographs of the cells showed honeycomb-like structures in these patterns (Figure 2f), with a width of approximately 1.5 µm (Figure 4c,d).

### 2.2. Ultrastructural Characterization of Tetraselmis jejuensis

**Figure 5:**
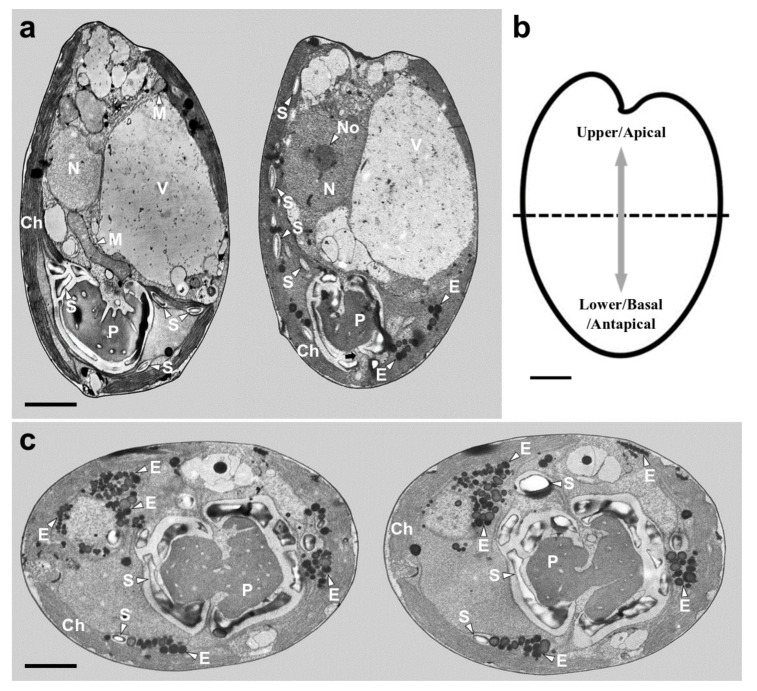
Micrographs of *Tetraselmis jejuensis* obtained using transmission electron microscopy. (**a**) Longitudinal sections showing several organelles inside the protoplasm, including chloroplasts (Ch), eyespot granules (E), mitochondria (M), nucleus (N), nucleolus (No), pyrenoid (P), starch (S), and vacuoles (V). The pyrenoid was invaded by both cytoplasmic channels comprising electron-dense material separated from the cytoplasm and canaliculi traversing it in opposite directions (black arrow). (**b**) Schematic drawing of longitudinal section indicating the upper/apical and lower/basal/antapical positions. (**c**) Transverse sections showing the position and structure of chloroplast (Ch), eyespot granules (E), pyrenoid (P), and starch (S). Scale bars = 2 µm.

**Figure 6:**
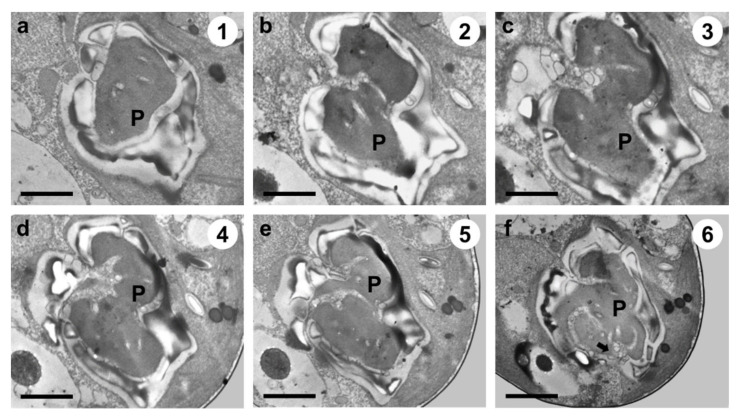
Serial TEM micrographs for the visualization of the pyrenoid matrix (P) of *Tetraselmis jejuensis*, which was penetrated by cytoplasmic channels (canaliculi) in various directions. The serial sections were obtained from the apical to the antapical direction in the cell. (**a**) The pyrenoid was encased in a starch sheath in the upper part. (**b–e**) It was invaded by canaliculi in the middle part. (**f**) Another penetration of cytoplasmic materials from a different direction was observed in the pyrenoid in the lower part (black arrow). The circled number in each micrograph indicates the section number in a series of sections. Scale bars = 1 µm.

TEM images of the longitudinal and transverse sections of *Tetraselmis jejuensis* cells showed the following cell organelles: a cup-shaped chloroplast enclosed in the outer plate membrane, a large vacuole, golgi apparatus, several starch granules, mitochondria, numerous eyespot globules, a pyrenoid, and a nucleus mostly observed on the left side near the upper part of the cell (Figure 5a,c). In the longitudinal sections of the cells, the pyrenoid was located in the basal position of the cell body and was embedded in the chloroplast (Figure 5a,b). The length and width of the pyrenoid in longitudinal sections were 1.3–4.0 µm (2.7 ± 1.0 µm), and 2.0–5.2 µm (3.6 ± 1.0 µm), respectively, measured by TEM micrographs (n = 20). The pyrenoid matrix of the cells was surrounded by a starch sheath, in which each granule showed a plate shape (Figure 5a,c). In both longitudinal and transverse sections of *T. jejuensis* cells, the matrix was invaded by cytoplasmic channels comprising electron-dense material separated from the cytoplasm and by small cytoplasmic channels (canaliculi) in various directions (Figure 5a,c). The large pyrenoid cavity opened towards the nucleus, but there were no intervening thylakoid membranes between the starch sheaths and the pyrenoid. These structures of the pyrenoid matrix were clearly visualized by serial TEM micrographs proceeding from the apical to the antapical region of the cells. In addition to a cavity invading the central pyrenoid, another cavity was observed in the matrix near the posterior region (Figure 6).

**Figure 7:**
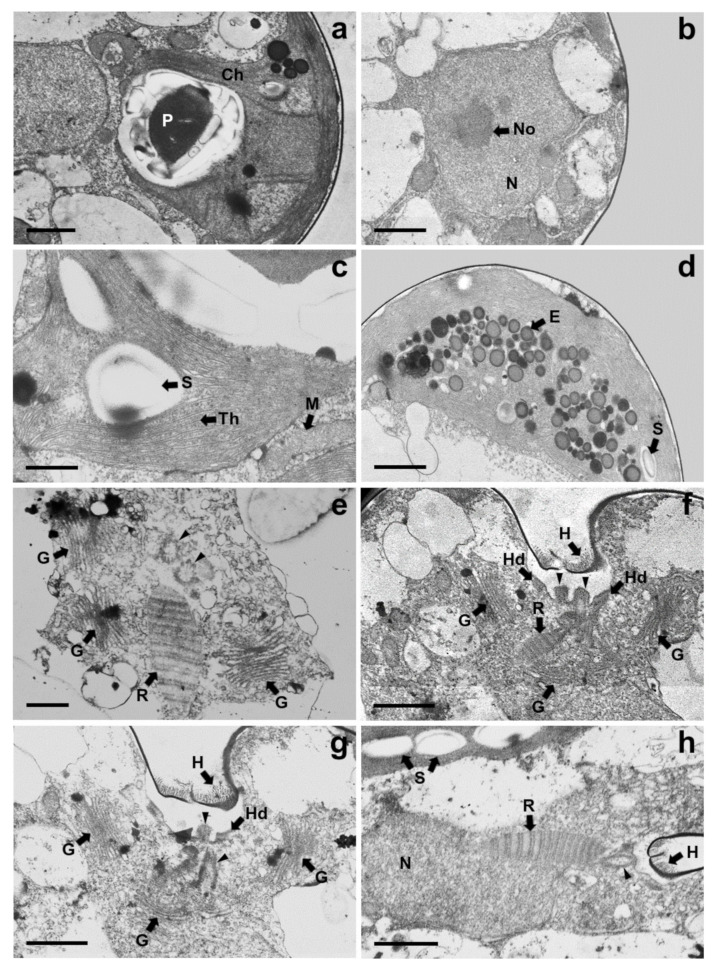
Magnified views of TEM micrographs of *Tetraselmis jejuensis* showing the position of cell organelles: (**a**) chloroplast (Ch) and pyrenoid (P); (**b**) nucleus (N) and nucleolus (No); (**c**) mitochondria (M), starch (S), and thylakoid membranes (Th); (**d**) Eyespot granules (E); (**e–h**) Flagellar apparatus of the cells; Flagella pit hairs (H), Golgi bodies (G), half-desmosome (Hd), rhizoplast (R), and basal bodies (black arrowheads). Scale bars = 1 µm for (**a**,**b**,**d**,**f**–**h**). Scale bars = 500 nm for (**c**) and (**e**).

Under TEM, magnified views of cellular ultrastructures showed that a pyrenoid was embedded in the chloroplast (Figure 7a); a round-shaped nucleus with a distinct nucleolus related to ribosome synthesis was encased by bilayer membranes and the nucleus was located near the flagellar base (Figure 7b,h); starch granules were sandwiched between thylakoid membranes of the chloroplast, and mitochondria were scattered throughout the cytoplasm in the cells (Figure 5a and 7c). A large number of eyespots (stigmas) were mostly positioned in the lower part of the cell body, especially near the antapical base. Each eyespot contained a large number of osmiophilic globules, which tended to be packed close together and were arranged in subcircular close packing of diverse sizes (Figure 5a,c and 7d). A cross-sectional image of the flagellar base showed a flagellar apparatus including the microtubule structure of the basal bodies, rhizoplast connecting the basal bodies to the nucleus, and Golgi bodies encircling the basal complex (Figure 7e,h). Notably, basal bodies connecting the flagellar filaments to the cell body were embedded in the flagellar apertures, and the slit width was approximately 0.23–0.34 µm (Figure 7f–h). Adhesive junctions, called half-desmosomes, which affix each flagellar basal body to the cell membranes, and flagella pit hairs located on the floor of the apical depression were also observed in the cells (Figure 7f,g).

### 2.3. Morphological Characterization of Tetraselmis jejuensis at Different Life Stages

**Figure 8:**
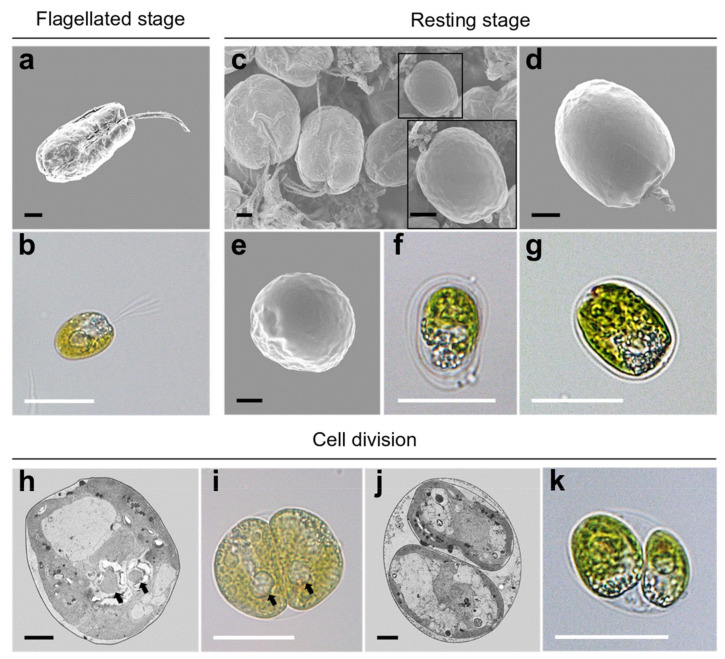
Morphological characteristics of *Tetraselmis jejuensis* in various life stages. (**a**,**b**) Motile cells, present in the flagellated stage. Resting cells were formed in response to stressful conditions or cellular reproduction. (**c**) Scattered cells including a resting cell highlighted by a black square, along with its magnified view. (**d**) Lateral, and (**e**) apical views of the resting cell. (**f**,**g**) Non-motile cells surrounded by single or bilayer membranes. (**h**–**k**) Asymmetric cell division in the process of vegetative reproduction; separated pyrenoids were observed (black arrows). Micrographs were obtained using SEM (**a**,**c**,**d**,**e**), TEM (**h**,**j**), and light microscopy (**b**,**f**,**g**,**i**,**k**). Scale bars (black) = 2 µm. Scale bars (white) = 20 µm.

During periods of unfavorable conditions or reproduction, flagellated cells (Figure 8a,b) are transformed into resting cells, and cysts are displayed in the resting phase of the cell cycle. In SEM micrographs, these resting cells were encapsulated by a smooth theca with a short papilla and lost their flagella (Figure 8c–e). Under light microscopy, a non-motile cell without the four flagella was enclosed with a single or bilayer membrane showing a very small aperture (Figure 8f,g). In the process of vegetative reproduction, *Tetraselmis jejuensis* cells underwent asymmetric cell division, resulting in morphological differences between daughter cells and heterogeneity within clonal cells. The two pyrenoids separated by cell division showed asymmetric properties (Figure 8h,i). The protoplasts of two daughter cells transversely divided in the parent cell wall were different in morphology and ultrastructure (Figure 8j,k).

### 2.4. Phylogenetic Analysis of Tetraselmis jejuensis

**Figure 9:**
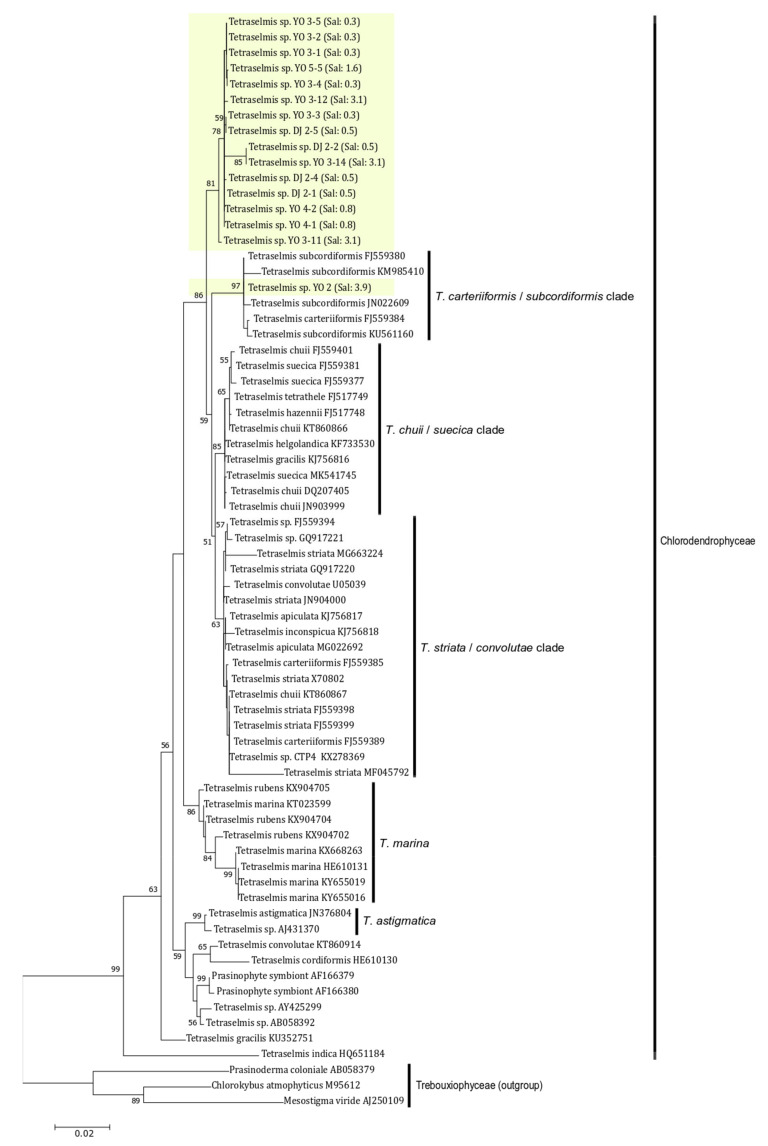
Phylogenetic analysis of *Tetraselmis jejuensis* using a Maximum-likelihood (ML) tree of the SSU rDNA sequences. Fifteen strains of *T*. *jejuensis* from this study formed a separate branch in the Chlorodendrophyceae except for one strain found at a salinity of 3.9%. ML bootstrap values (>50) are shown next to the branches. Scale bar = the number of nucleotide substitutions per site.

The SSU rDNA sequences of 16 *Tetraselmis jejuensis* strains were determined by PCR amplification, and ranged from 1514 to 1631 bp. We investigated phylogenetic relationships based on SSU rDNA sequences between *T*. *jejuensis* and other *Tetraselmis* species deposited in GenBank. A maximum likelihood (ML) tree for 70 candidates showed that the 16 isolated strains of *Tetraselmis* species were positioned in the class Chlorodendrophyceae (Figure 9). Of these, 15 strains formed a newly branched clade, with 86% bootstrap value in the class. Only the *Tetraselmis* sp. YO 2 strain, found at a salinity of 3.9%, was included in the *Tetraselmis carteriiformis/subcordiformis* clade, with a 97% bootstrap value. For phylogenetic relationships based on the ML tree of SSU rDNA, the isolated strains in this study were relatively closer to the *Tetrasemis carteriiformi/subcordiformis* clade, *Tetrasemis chuii/suecica* clade, and the *Tetrasemis striata/convolutae* clade than to the early diverging clades in the family, such as *Tetraselmis marina*, *Tetraselmis astigmatica*, *Tetraselmis indica*, and others.

## 3. Discussion

Supralittoral tide pools made of basalt on the Jeju Island coastline have varying salinities, ranging from 0.1–10%. These extreme changes in salinity in these tide zones are caused by the precipitation, evaporation, and varying environmental conditions on Jeju Island. In these areas, rain pools with very low salinity levels existed in the area near the land, and the pool near the sea had a salinity similar to that of seawater. A salinity gradient had occurred between the two regions, and sometimes the pool with a very high salinity was formed by evaporation. Therefore, the salinity of each pool varied depending on rainfall, evaporation, and waves, but the salinity of the rain pools existing near the land, which the breaking waves could not influence, were maintained relatively low.

In this study, we found the euryhaline microalga, *Tetraselmis jejuensis*, in two supralittoral tide pools on Jeju Island: Yongduam (YO) in the north, and Daejeong in the south. Collected cells were found to naturally survive in the supralittoral zones with a wide salinity range from 0.3–3.1% (Figure 11 and Table 3). Tetraselmis *suecica* and *Tetraselmis tetrathele*, which are widely known euryhaline species, are used as feedstock, and require salinities of 2.0–6.0% and 3.0–3.5% for their growth, respectively [38,39]. *Tetraselmis* sp. CTP4 strains could be cultivated at salinities ranging from 0.5–3.6% [13], and the optimal growth of *Tetraselmis gracilis* strains found on the Jeju coast with regard to salinity changes ranged from 2.0–2.5% [28]. However, robustness to various salt concentrations was evaluated by the manipulation of salinity in the laboratory-cultured medium. Thus, the physiological characteristics of *T*. *jejuensis* are distinct compared to those of other *Tetraselmis* species reported previously, and an optimal salinity of less than 1.0% for the growth of *Tetraselmis* species has been rarely reported. The morphological characteristics of the genus *Tetraselmis* have been well studied by many researchers for taxonomic identification [22,31,32,33,34,35,40]. The new species, *T*. *jejuensis*, shares these taxonomic characteristics with the genus *Tetraselmis.* Its cells are elliptically shaped and covered by a scale-based theca with four emerging equal flagella from the apical depression. The nucleus lies close to the flagellar apparatus, and a cup-shaped chloroplast includes eyespot regions and one pyrenoid inside the cell protoplasm. The pyrenoid is surrounded by starch and is embedded in the chloroplast.

Comparing the morphological and ultrastructural features between *T. jejuensis* and other *Tetraselmis* species reported in previous studies, we found several characteristics of *T. jejuensis* by light microscopy, SEM, and TEM (Table 1). First, distinct creases were observed in the middle of the cell body in both the broad and narrow lateral views (Figure 1c and Figure 2a–f). As previously reported by [22,31], these creases are not key for classifying the species of *T. jejuensis*. However, micrographs clearly showing these furrows on the cell surface have been rarely presented so far, including the above results obtained using light microscopy and a vaguely visualized SEM micrograph, respectively. According to [41], *Tetraselmis indica* cells contain creases that divide the cell into longitudinal sections, but these creases are observed only in the broad face of the cells. Second, some scale particles were particularly spread out on the surface at the antapical side of the cells (Figure 2e,f). Recently, [41] reported that these kinds of cells are observed on the cell surface of *Tetraselmis indica*, with a maximum scale size of 1.08 µm, in agreement with the measurements in this study. However, these shapes and sizes varied between different species; *T. indica* particles exhibited a hollow rim shape, whereas those of *T*. *jejuensis* showed a shallow dent-like structure, with a maximum depth of 0.58 µm in the cells. Notably, we found, by SEM, distinct patterns containing honeycomb-like structures in the middle of the broad lateral side of *T*. *jejuensis* (Figure 2a,f and Figure 4). It is thus important to determine whether *Tetraselmis* species with or without these patterns belong to *T*. *jejuensis*, because this pattern has rarely been reported so far. It would thus be worthwhile to examine whether other *Tetraselmis* species have such honeycomb-like structures.

In [34], the authors proposed that the genus *Teraselmis* should be divided into four subgenera (*Tetraselmis*, *Prasinocladia*, *Tetrathele*, and *Parviselmis*) based on the ultrastructural features of the pyrenoid matrix, which is the ellipsoidal protein matrix that mediates efficient carbon fixation with the enzyme Rubisco, as visualized by TEM [35,43,44]. In Chile, several strains of *Tetraselmis* that were difficult to be distinguished as different species when using light microscopy, could be classified according to the criteria suggested by [34,45]. Two of them belonged to the subgenus *Parviselmis*, whereas the others belonged to the subgenus *Tetrathele*. By tracing these pyrenoid characteristics established as ultrastructural markers, we investigated the ultrastructures of *T*. *jejuensis* using TEM. The pyrenoid of the cell was surrounded by a starch sheath, wherein each starch granule was plate-shaped. The pyrenoid was invaded by several cytoplasmic channels in various directions with the presence of a major cavity that opened towards the nucleus, indicating that the species *T*. *jejuensis* belongs to the subgenus *Tetrathele* (Figure 5a,c and Figure 6). It is distinct from other *Tetraselmis* species belonging to the subgenera *Tetraselmis* (*T*. *cordiformis*, *T*. *ascus*, *T*. *convolutae*, *T*. *astigmatic*), *Prasinocladia* (*T*. *marina*, *T*. *verrucosa*, *T*. *rubens*), and *Parviselmis* (*T. levis*, *T. chuii*, *T. suecica*, *T. alacris*, *T. striata*) [34,35,46].

**Figure 10:**
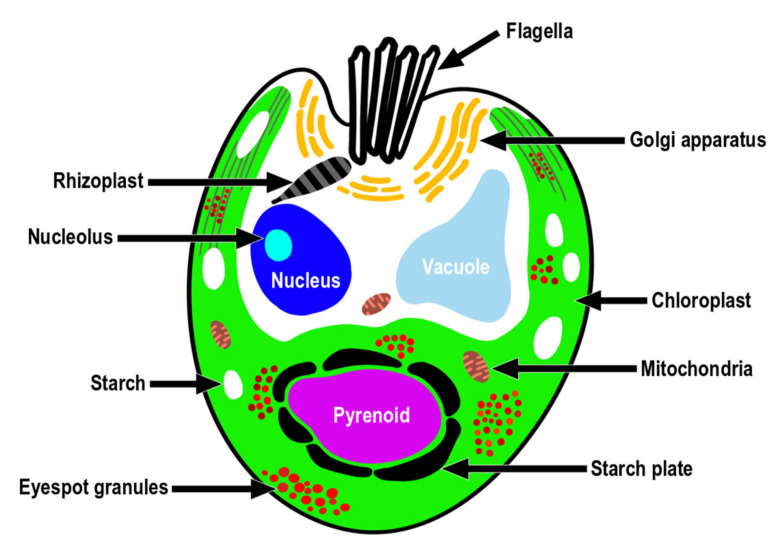
A schematic drawing of the ultrastructure indicating the position of the major cell organelles in *Tetraselmis jejuensis*.

Consistent with the above results, the ultrastructural features of *T*. *jejuensis* are different from those of other *Tetraselmis* species. First, the location of the pyrenoid matrix in the body of *T*. *jejuensis* is lower than that of other species, including *T. alacris*, *T. ascus*, *T. chuii*, *T. indica*, *T. levis*, and *T. verrucosa* [31,34,35,41] (Figure 5a,c and Figure 6 and Table 1). Second, the eyespot (stigma) of *Tetraselmis* species is commonly apart from flagellar roots as a specific feature, whereas that in *Chlamydomonas reinhardtii*, with a well understood flagellar assembly, is located near the microtubule roots [33,47,48]. In TEM micrographs of *T*. *jejuensis*, numerous eyespot globules were observed in the lower part of the cell body, especially near the antapical base (Figure 5a,c). By light microscopy, numerous orange-red eyespot regions were found to be dispersed throughout the chloroplasts of *T*. *jejuensis* cells (Figure 1d–g). Notably, the number of eyespots in the *T*. *jejuensis* cell body was much higher than that previously reported in all other *Tetraselmis* species (Table 1). Most *Tetraselmis* species contain a single conspicuous eyespot region composed of one or two layers of osmiophilic granules in the cells [22,31,32,33,34,35]. Moreover, the eyespots of some species, such as *T*. *alacris*, *T*. *astigmatica*, *T*. *levis*, and *T*. *suecica*, were not present or were not conspicuous inside the protoplasm. Recently, Arora et al. [41] confirmed that *Tetraselmis indica* cells contain one or several eyespots below the pyrenoid. However, the number of eyespot globules in *T*. *indica* was far less than that in *T*. *jejuensis*. Melkonian & Robenek discussed the possible role of the eyespot in *Tetraselmis* phototaxis as well as structural features that may indicate its importance to cell orientation toward light [49]. Since *T. jejuensis* was found in a shallow tidal pool that was affected by direct sunlight, it is thought that strong light influences the development of the eyespot. Thus, these distinctive features in the ultrastructure of *T*. *jejuensis* can play an important role in determining whether unidentified species could be *T*. *jejuensis*. The ultrastructural profile of *T*. *jejuensis* with the major cellular organelles is schematized in Figure 10.

Further investigation of the similarity between these cells and three Chilean strains of *Tetraselmis* reported by Gonzalez et al. [45] revealed that the ultrastructural arrangement of *T*. *jejuensis* is similar to that of the Dichato strain, despite the structures of the pyrenoid matrix differing. Although the environmental data for Coliumo Bay, where Dichato is located, was not provided at the time, we confirmed that the salinity of Coliumo Bay was approximately 1.36%, measured at depths ranging from 2 m to 5 m in November 1996 [50]. The finding that these cells proliferate vigorously at salt concentrations lower than 2.0% corresponds to the physiological characteristic of *T*. *jejuensis* surviving in extreme salt concentrations ranging from 0.3% to 1.6% (Table 3). Unfortunately, comparison of aligned sequences between these strains is limited by the absence of deposited SSU rDNA sequences of the Chilean strains in GenBank [45].

The flagellar root system of *Tetraselmis* species has been well studied and established in previous studies [22,33,34,35,41]. It has been reported that Golgi bodies are commonly located around the flagellar base in *Tetraselmis* species because they are associated with flagellar regeneration via their activation [51,52]. The Golgi apparatus of *T*. *jejuensis* was found to be present around the basal complex surrounding them (Figure 7e–g). Furthermore, the structures of flagellar apparatus in the cells, including rhizoplasts, basal bodies, half-desmosomes, and flagella pit hairs on the apical floor are equivalent to those of other *Tetraselmis* species, as reported previously.

To clarify the phylogenetic relationships between a newly branched clade containing *T*. *jejuensis* and other clades classified within the genus *Tetraselmis*, we compared the SSU rDNA sequences of 16 isolated *T*. *jejuensis* strains with those of the identified species in each clade, based on the number of base pairs that were different from each other. Four strains isolated from Daejeong (DJ) displayed 1.53% similarity to *T. suecica* (MK541745), with 1.59%, 1.78%, 1.85%, and 2.28% dissimilarity to *T. chuii* (DQ207405), *T. subcordiformis* (FJ559380), *T. carteriiformis* (FJ559384), and *T. convolutae* (U05039) as indicated for maximum value, respectively, indicating that the *T*. *jejuensis* DJ strains within the newly branched clade were 1.53% different from *T. suecica* (MK541745), which is the closest species in the Chlorodendrophyceae, based on the phylogenetic tree of SSU rDNA (Table 2). Next, we confirmed that 12 strains isolated from YO showed 1.19% dissimilarity to *T. suecica* (MK541745), with 1.39%, 1.55%, 1.61%, and 2.11% dissimilarity to *T. chuii* (DQ207405), *T. subcordiformis* (FJ559380), *T. carteriiformis* (FJ559384), and *T. convolutae* (U05039) as indicated for the maximum value, respectively. Exceptionally, a strain (YO 2) found at a salinity of 3.9% showed 0.13% dissimilarity to *T. subcordiformis*, with 0.19%, 1.26%, 1.32%, and 1.75% similarity to *T. carteriiformis*, *T. suecica*, *T. chuii*, and *T. convolutae*, respectively. These results indicated that *T*. *jejuensis* YO strains were 1.19% different from *T. suecica* (MK541745), which is the closest species in the phylogenetic analysis, except for the YO 2 strain, which is analogous to *T. subcordinormis* (Table 2). Hence, a distinctive clade containing the DJ and YO strains within the Chlorodendrophyceae is considered a new species, based on the phylogenetic tree of SSU rDNA sequences.

Consistent with the phylogenetic analysis, the ultrastructural details of *T*. *jejuensis* were different from those of *T. suecica*, its closest species. *T*. *jejuensis* cells had numerous eyespot globules and belonged to the subgenus *Tetrathele*, whereas *T*. *suecica* cells were characterized by non-conspicuous eyespots and belonged to the subgenus *Parviselmis*. Moreover, according to Fabregas et al. [53], the optimal salinity for growth in *T*. *suecica* was between 2.5% and 3.5%; Pugkaew et al. [39] also confirmed that the optimal salinity range of *T. suecica* for growth was 2.0%–6.0%, whereas significant growth inhibition was observed at a low salt concentration (1.0%). However, *T*. *jejuensis* DJ and YO strains isolated from supralittoral tide pools on Jeju Island actively thrive and grow in their habitats with salinities of 0.3%, 0.5%, 0.8%, 1.6%, and 3.1%, respectively (Table 3). Given the differences based on phylogenetic, ultrastructural, and physiological features, T. jejuensis is distinct from *T. suecica*.

## 4. Materials and Methods

### 4.1. Sample Collection and Strain Setup

**Figure 11:**
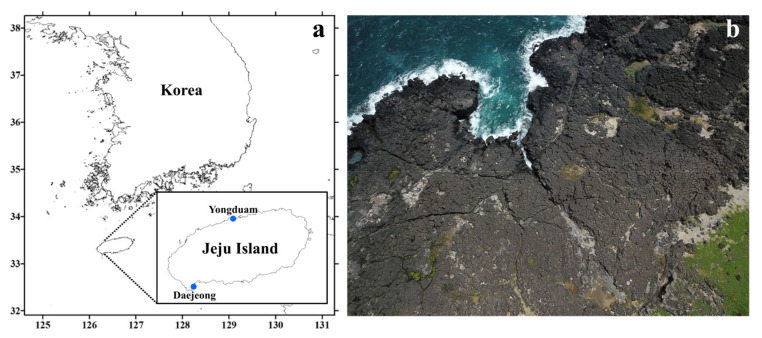
Location and aerial photographs of the sampling sites in Jeju Island. (**a**) Map of the sampling sites; location of Daejeong and Yongduam sites in Jeju Island. (**b**) The images show supralittoral tide pools made of basalt on the coastline of Jeju Island, Korea; images acquired using an unmanned aerial vehicle (UAV).

Microalgal samples containing *Tetraselmis* species were collected from two supralittoral tide pools, Daejeong (33.2126, 126.2948) and Yongduam (33.5159, 126.5120), on Jeju Island in April and June 2019 (Figure 11, Table 3). For getting samples with various salinity concentrations from 0% to 4%, sampling was performed 5 days after rainfall. The salt concentrations of samples from Daejeong (DJ), located in the south of Jeju, varied from 0.5% to 2.6%, and those from Yongduam (YO) in North Jeju ranged from 0.3% to 4.4%, because the salinities of these tide depressions were different at the time of sampling. The average temperatures of DJ and YO at the sampling time were 21.3 °C and 25.1 °C, respectively. Live samples were transferred to the laboratory, and single cells were isolated in six-well plates using a dissecting microscope (SZX10, Olympus, Tokyo, Japan). Clonal cultures of *T*. *jejuensis* were established using two serial single-cell isolations. Cells were grown in f/2 culture medium [54] at 22 °C, with continuous illumination at 65 μmol photons/m^2^/s, and optimal salinities of 0.3, 0.5, 0.8, 1.6, 3.1, and 3.9% based on the salt concentrations of each collected sample (Table 3). After sufficient growth, the clonal cultures were transferred to 30 mL flasks and 200 mL PC bottles. Sixteen strains of *T. jejuensis* (4 strains from DJ, and 12 strains from YO) were established, up to a cell density of at least 5.0 × 10^4^ cells/mL.

### 4.2. Microscopy

Living cells were observed under a light microscope, and their length and width were measured using an Olympus BX 53 microscope equipped with a DP73 digital camera system (Olympus, Tokyo, Japan). For field emission scanning electron microscopy (FE-SEM: Sigma 500/VP, Carl Zeiss, Oberkochen, Germany), 10 mL aliquots of cultures at approximately 2 × 10^6^ cells mL^−1^ were fixed for 10 min in osmium tetroxide (OsO_4_, Electron Microscopy Sciences, Hatfield, PA, USA) at a final concentration of 1% (*v*/*v*). The fixed cells were collected on 3 µm pore size polycarbonate membrane filters (Whatman, Kent, UK) and washed thrice with 50% filtered seawater diluted with distilled water to remove residual salts. The membranes with attached cells were dehydrated in an ethanol series (10, 30, 50, 70, 90, and 100% ethanol, followed by two changes in 100% ethanol (Merck, Darmstadt, Germany), and were immediately dried using an automated critical point dryer (EM CPD300, Leica, Wetzlar, Germany). The dried filters were mounted on an aluminum stub (Electron Microscopy Sciences, Hatfield, PA, USA) using copper conductive double-sided tape (Ted Pella, Redding, CA, USA), and coated with gold using an ion sputter (MC1000, Hitachi, Tokyo, Japan). The cells and surface morphologies were observed using a high resolution Zeiss Sigma 500 VP field-emission scanning electron microscope (FE-SEM, Sigma 500/VP, Carl Zeiss, Oberkochen, Germany). The apical depression width, width of specific scales, regular subunit patterns on the cell surface, and thin pit hair length of *T*. *jejuensis* cells were measured using SEM micrographs (SmartSEM version 6.08, Carl Zeiss, Oberkochen, Germany). For transmission electron microscopy (TEM), cells were transferred to a 10 mL tube and fixed in 2.5% (*v*/*v*) glutaraldehyde (final concentration) for 1.5 h. Tube contents were placed in a 10 mL centrifuge tube and concentrated at 1,610 × *g* for 10 min in a Vision Centrifuge VS-5500 (Vision, Bucheon, Korea). The resulting pellet was subsequently transferred to a 1.5 mL tube and rinsed in 0.2 M sodium cacodylate buffer (Electron Microscopy Sciences, Hatfield, PA, USA) at pH 7.4. After several rinses in 0.2 M sodium cacodylate buffer, cells were post-fixed for 90 min in 1% (*w*/*v*) OsO_4_ in deionized H_2_O. The pellet was then embedded in agar. Dehydration was performed in a graded ethanol series (50, 60, 70, 80, 90, and 100% ethanol, followed by two changes in 100% ethanol). The material was embedded in Spurr’s resin (Electron Microscopy Sciences, Hatfield, PA, USA). Sections were prepared using an EM UC7 ultramicrotome (Leica, Wetzlar, Germany) and stained with 3% (*w*/*v*) aqueous uranyl acetate (Electron Microscopy Sciences, Hatfield, PA, USA) followed by lead citrate (Electron Microscopy Sciences, Hatfield, PA, USA). The sections were visualized using Sigma 500/VP TEM (Sigma 500/VP, Carl Zeiss, Oberkochen, Germany). The length and width of the pyrenoid and the slit width of the embedded basal bodies of *T*. *jejuensis* cells were measured using TEM micrographs (SmartSEM version 6.08, Carl Zeiss, Oberkochen, Germany).

### 4.3. DNA Extraction and PCR Amplification

We prepared 2 mL of *Tetraselmis jejuensis* cultures with a density of 1.0 × 10^5^ cells/mL, collected cell pellets by centrifugation at 19,745× *g* for 3 min, and stored these at −80°C until DNA extraction. In brief, we extracted total genomic DNA (gDNA) from the preserved pellets using the AccuPrep Genomic DNA Extraction Kit (BIONEER, Daejeon, Korea), following the manufacturer’s instructions. The rDNA was then amplified using universal eukaryotic primers [55,56,57,58,59]. The reaction mixtures for PCR amplification contained 5 µL of 10X F-Star Taq Reaction Buffer with 1 µL of 10 mM dNTP mix, 0.02 μM of primers, 0.25 μL of 5 U/μL BioFACT™ F-Star Taq DNA polymerase (BioFACT Co., Ltd., Daejeon, Korea), 38.75 µL of UltraPure™ DNAse/RNAse-Free Distilled Water (Invitrogen, Carlsbad, CA, USA), and 3 µL of DNA template (ca. 10–30 ng of DNA concentration). PCR amplification was conducted using an Eppendorf Mastercycler PCR machine (Eppendorf, Hamburg, Germany) under the following thermal cycling conditions: pre-denaturation at 94 °C for 5 min, followed by 40 cycles of 95 °C for 30 s, the selected annealing temperature (AT) for 30 s, 72 °C for 1 min, and a final extension at 72 °C for 10 min. The AT of the primers was determined using gradient PCR. We optimized the AT as follows: 56.0 °C (EukA-G18R), 56.0 °C (570F-EukB), 60.0 °C (EukA-EukB), 58.0 °C (ITSF2-LSUB), 56.0 °C (ITSF2-LSU500R), and 56.0 °C (Euk1209F-1483R). Sequences of the universal primers were specified in Table 4. PCR products were purified using AccuPrep PCR Purification Kit (BIONEER, Daejeon, Korea) and subjected to Sanger sequencing provided by BIONEER service, and the obtained nucleotide sequences were identified using BLAST in the National Center for Biotechnology Information (NCBI) database.

### 4.4. Phylogenetic Analysis

The test species based on similarities in phylogenetic analysis were obtained from GenBank and related studies [13,41]. Seventy SSU rDNA sequences from the class Chlorodendrophyceae were included, which contained 16 isolated strains of *Tetraselmis* species from this study and 51 relatives, as well as Trebouxiaceae, from which three species were used as outgroups. These sequences were aligned using the SILVA alignment service [60]. A phylogenetic tree of aligned sequences was constructed using the maximum likelihood (ML) algorithm in MEGA7 [61]. ML bootstrap values were calculated using 1000 replicates from the same substitution model.

## 5. Conclusions

We found euryhaline microalga, *Tetraselmis jejuensis*, adapted to supralittoral tide pools on Jeju Island, Korea, in which salinities ranged from 0.3% to 3.1%. For taxonomic identification, isolated strains from DJ and YO were examined by light microscopy, SEM, TEM, and phylogenetic analysis based on SSU rDNA sequences. First, the cells have specific scales and a regular subunit pattern, with a honeycomb-like structure, on the cell surface. Second, the pyrenoid structures of the cells belong to the subgenus *Tetrathele*, whereas those of most other *Tetraselmis* are included in the subgenera *Tetraselmis*, *Prasinocladia*, and *Parviselmis*. Notably, a large number of eyespot globules are dispersed throughout the chloroplast, especially at the posterior part of the cell body. For phylogenetic relationships, both DJ and YO strains constitute a newly branched clade in Chlorodendrophyceae based on SSU rDNA sequences. Each of the strains shows 1.53% and 1.19% dissimilarity with *T. suecica*, the closest species, respectively. Fifteen strains, including the separate clade, were regarded as the same species. The physiological features of these strains surviving under natural salinity conditions ranging from 0.3% to 3.1% are distinct compared to those of the other *Tetraselmis* species. To our knowledge this is the first report of *Tetraselmis* species living in supralittoral tide pools on Jeju Island, Korea. These results led us to propose the isolated species showing optimal growth in salinities of 0.3% to 3.1% as *Tetraselmis jejuensis*.

## Taxonomic Summary

Phylum Chlorophyta [62]

Class Chlorodendrophyceae [17]

Order Chlorodendrales [16]

Family Chlorodendraceae [63]

*Tetraselmis jejuensis* sp. nov.

Diagnosis: Cells are compressed and elliptical in shape. The anteroposterior (AP) length, width, and depth of the cells in the broad lateral view measured by light microscopy were 13.0–20.8 µm, 6.5–16.3 µm, and 9.8–13.0 µm, respectively. The cells are covered by a scale-based theca with four equal flagella emerging from an apical depression in two opposite pairs. There are four distinct creases on the cell surface. Each deep-folded line on the broad or narrow lateral sides of the cell is carved in the middle of the cell body and extends longitudinally from the anterior to the posterior of the cells. Specific scales are spread over the cell surface, especially at the antapical side. In the broad lateral views of the cells, there is a unique, regular subunit pattern with honeycomb-like structures on the surface at the posterior part near the crease. The nucleus located in the upper part of the cell body is close to the flagellar apparatus. The yellow-green, cup-shaped chloroplast contains a pyrenoid and numerous eyespot globules. The pyrenoid is located in the lower part of the cell body adjacent to the basal position; the length and width of the pyrenoid in the longitudinal view are 1.3–4.0 µm (2.7 ± 1.0 µm), and 2.0–5.2 µm (3.6 ± 1.0 µm), respectively, as measured by TEM. The pyrenoid matrix is invaded by a major cavity filled with cytoplasmic channels and several canaliculi in various directions, and is surrounded by starch in a plate shape. Numerous orange-red eyespot globules are mostly located in the lower part of the cell body, especially near the basal position, and are dispersed throughout the chloroplast. Two to four Golgi bodies encircle the basal complex around the flagellar base. The cells undergo asymmetric cell division, and the daughter cells show morphological and ultrastructural differences within clonal cells.

Type locality: Daejeong, Jeju Island, Korea (33°12'45.4" N, 126°17'41.4" E), Yongduam, Jeju Island, Korea (33°30'57.2" N, 126°30'43.3" E).

Etymology: The specific name “jejuensis” refers to the name of the collection site on Jeju Island, Korea.

## Holotype

The SEM stub containing the type material from strain XXXXXX was deposited at the collection of the National Marine Biodiversity Institute of Korea (MABIK), Seocheon, South Korea, under accession number MABIKfl00030738. Gene sequence: DNA sequences obtained from clonal strains of *Tetraselmis jejuensis* were deposited in GenBank under accession no. MZ435987, MZ435988, MZ435989, MZ435990, MZ43591, MZ435992, MZ435993, MZ435994, MZ435995, MZ435996, MZ435997, MZ435998, MZ435999, MZ436000 and MZ436001.

## Figures and Tables

**Table 1:** Comparison of morphological and ultrastructural characteristics between *Tetraselmis jejuensis* and other *Tetraselmis* species reported previously. Several species were described before the emergence of electron microscopy. ND, information not available.

Species	Location and Type of Habitat	Cell Shape and Size	Regular Subunit Pattern	Specific Scales on the Cell Surface	Pyrenoid Matrix and Subgenus	Eyespot (Stigma)	Nuclear Position	Chloroplast	Golgi Bodies	References
*Tetraselmis jejuensis*	Jeju Island, Korea; supralittoral tide pools	Compressed, elliptical, and four creases obviously showing in both broad and narrow lateral views; 13.0–20.8 × 6.5–16.3 × 9.8–13.0 µm	Observed, honeycomb-like structure	Observed, a shallow dent-like shape, measuring a maximum of 0.58 µm	Present in the lower part of the cell body adjacent to the antapical base, surrounded by starch plates; *Tetrathele*	Numerous eyespot globules, mostly located on the lower part of the cell body and spread out in the chloroplast	Located in the upper part of cell body near the flagellar base	Cup shaped, enclosed by the outer plate membrane	2–4, around the flagellar base	This study
*T. alacris*	Europe and North America; rock pools	Compressed, broadly ovoid; 9.5–12 × 8.0–8.5 × 6.5–7.0 μm	ND	ND	Small, spheroidal, central, surrounded by concave-convex starch grains; *Parviselmis*	Inconspicuous, located around the pyrenoid	Central	Finely lobed in the posterior	2, around the flagellar base	[31,35]
*T. apiculata*	France; estuaries	Slightly compressed, broadly elliptical to narrowly oval, 7.5–10.5 × 6.5 × 4.5–5 μm	ND	ND	Basal, spherical	Single, located in the upper part of the cell	ND	Deeply bilobed at the anterior end	ND	[31]
*T. ascus*	Pacific coast of North America and Japan; small tide pools	Elliptical, 19–30 × 8–16 μm	ND	ND	Large, circular, located near the center of the cell, surrounded by lens-shaped starch grains; *Tetraselmis*	Located in the anterior third of the chloroplast, 1.5 to 2.0 µm indiameter, a single region composed of two or three layers of lipid granules	ND	Four lobes in the anterior	5, surrounding the basal body	[34]
*T. astigmatica*	Pacific coast of North America; salt marsh	Spherical, 11–19 × 7–16 μm	ND	ND	Large, located in the posterior part of the cell surrounded by lens-shaped starch grains; *Tetraselmis*	Not present	ND	Large, invaginated with cytoplasmic canaliculi in the posterior	4, surrounding the basal body	[34]
*T. carteriiformis*	Scotland; rock pools	Compressed, ovoid in front view, 12–14 × 9–10 × 7–8 μm	ND	ND	Large, basal, irregularly rounded, with a starch sheath	Single, sub-median, in the region of the pyrenoid	Central	Narrowly four-lobed, in the region of the pyrenoid	ND	[31]
*T. chuii*	Europe and North America; tide pools and estuaries	Compressed, elliptical to obovate, 12–16 × 7–10 μm	ND	ND	Small, irregular in shape, located far from the nucleus surrounded by concave-convex starch grains	Conspicuous, a single region composed of two layers of osmiophilic granules, 1.3–2.5 µm in size, located usually in the upper region of the pyrenoid, but variable in position	At the anterior half of the cell body	Finely lobed	2, around the flagellar base	[35]
*T. contracta*	UK; marine	Compressed, broadly elliptical, 25 × 17 × 11 μm	ND	ND	Oval, medium, basal	Single, conspicuous, central to anterior	ND	Two large and two small apical lobes	ND	[31]
*T. convolutae*	Europe and Japan; marine	Compressed, shape variable, occasionally curved, 8–13 × 6–10 × 4–6 μm	ND	ND	Conspicuous, 2–4 μm, in the posterior, appearing eccentric with ring-shaped starch	Exceptionally large, 1–2.3 μm, located in the anterior third of the body, a single region composed of two layers of osmiophilic granules	Central	Four lobes extending forward from just behind the middle of the body	2–4, around the flagellar base	[32]
*T. cordiformis*	Freshwater	Compressed, obovate, 17–19 × 13–16 × 8–11 μm	ND	ND	Large, located directly beneath the nucleus surrounded by biconvex-shaped starch grains; the matrix penetrated from all directions with canaliculi	Located near the middle of one of the broad sides, but considerably variable in position, approximately 1.5 μm in diameter	ND	Large, highly reticulate in the posterior	2–4, around the flagellar base	[33,34]
*T. gracilis*	Europe; marine	Compressed, broadly to narrowly elliptical, 8–9 × 5.5–7.5 × 5–6.5 μm	ND	ND	Large, spherical, sub-basal, with a U-shaped starch sheath	Single, conspicuous, situated in the anterior half of the cell	ND	Uniformly and markedly rugose, axile	ND	[31]
*T. hazeni*	Europe: Spain, and USA; marine	Compressed, elliptical to oval, 13–17 × 7–8 × 4–5 μm	ND	ND	Basal, cup shaped, rather large	Small, single, situated in the upper part of the pyrenoid	ND	Cup shaped, with 4 anterior lobes but non-posterior	ND	[31]
*T. helgolandica*	Helgoland; marine	Compressed, oval, 21–24 × 14–15 × 7–9 μm	ND	ND	Conspicuous, spherical, sub-central to sub-basal, with large starch grains	3–6	ND	A shorter posterior lobe, and two longitudinal lateral lobes	ND	[31]
*T. impellucida*	Puerto Rico; marine	Slightly compressed, shape variable, 14–23 × 8–17 μm	ND	ND	Absence of a starch sheath around the pyrenoid, lying posterior to nucleus	Conspicuous, composed of two layers	Located subapically below the apical trough	Cup shaped, covering the peripheral region with a slit from the cell apex to the middle of the body	ND	[42]
*T. inconspicua*	Europe; marine	Slightly compressed, oval in front, elliptical in lateral view, 4.5–7 × 4.5–6 × 3.5–4 μm	ND	ND	Basal, very small, globular, with a continuous starch sheath	Single, conspicuous, in the region of pyrenoid	ND	Anterior two lobed to the center of the cell	ND	[31]
*T. indica*	India (Goa); hypersaline to marine	Slightly compressed, elliptical, and a folded line faintly observed in the broad lateral views; 10–25 × 7–20 × 6.5–18 μm	ND	Observed, a hollow rim-like shape	Central, hardly observed starch plates	Conspicuous, one or sometimes several, situated below the pyrenoid	Present in the anterior half of the cell	Cup shaped with 4–8 lobes	2–8, around the flagellar base	[41]
*T. levis*	England; salt marsh	Compressed, ovate, 9–12 × 6–7.5 μm	ND	ND	Irregular in shape, located sub-centrally, surrounded by biconvex starch grains; *Parviselmis*	Not conspicuous, located in the region of the pyrenoid	Near the flagellar base	Finely lobed in the posterior end	2	[35]
*T. maculata*	Europe, collected from salt marsh pools and apparently not common; marine	Slightly compressed, ovate in front, elliptical in lateral view, 8–9 × 5.5–7.5 × 5–6.5 μm	ND	ND	Basal, medium, or small, with a discontinuous starch sheath	Single, conspicuous, close to the pyrenoid	ND	Finely granular, anterior two lobes	ND	[31]
*T. marina*	Europe, North America and Japan; marine	Plants unicellular or colonial with a septate stalk, cells elliptical, 16–20 × 7–8 μm	ND	ND	Large, almost spherical, with concave starch grains	Conspicuous, a single region composed of two layers, located peripherally at a level between the nucleus and pyrenoid	ND	Massive, cup shaped, located peripherally with 4 anterior lobes	5, near the anterior end of the nucleus	[22]
*T. rubens*	Europe; marine	Compressed, 8–11 × 5–8 × 4.5–5 μm	ND	ND	Basal, medium, globular with a U-shaped starch sheath	Single, conspicuous, in the anterior to the middle	ND	Anterior deeply 2 lobed	ND	[31]
*T. striata*	Europe and Japan; brackish water and tide pools	Compressed, elliptical, 7–11 × 5.5–7.2 μm	ND	ND	Small, located sub-basally, surrounded by biconvex starch grains; *Parviselmis*	Conspicuous, a single region composed of one or two layers, larger than the pyrenoid matrix, 1.7–3 µm in diameter, located lateral to the pyrenoid in the posterior half	Central	Dorsiventrally lobed into two posterior sections	2	[35]
*T. subcordiformis*	Norway; marine	Compressed, elliptical, 11–17 × 8–10 μm	ND	ND	Large, spherical, sub-central to sub-basal	Single, in lower part of the cell near the pyrenoid	ND	A shorter posterior lobe and two lateral lobes	ND	[31]
*T. suecica*	Brackish, marine	Compressed, elliptical to obovate, 6–11 × 4–8.5 μm	ND	ND	Spheroidal, located near the base, surrounded by concave-convex shaped starch grains; *Parviselmis*	Not conspicuous, located near the pyrenoid	Central	Cup shaped, usually simple, rarely bilobed in the posterior part	2	[35]
*T. tetrathele*	Europe; marine	Compressed, elliptical, 10–16 × 8–11 × 4.2–5 μm	ND	ND	Large, spherical, sub-central to sub-basal	Single, sub-median, usually situated in the region of the upper part of the pyrenoid	ND	Axile with a narrow sinus reaching the pyrenoid, a shorter posterior lobe and two lateral lobes	ND	[31]
*T. verrucosa*	Europe and Japan;brackish water	Compressed, elliptical in front view with a deep apical furrow in lateral view, 8.5–10 × 6–6.5 × 4.5–6 μm	ND	ND	Small, spherical, sub-basal or central, with a starch sheath	Single, conspicuous, and variable in position	Scattered irregularly	Massive, with 2 anterior lobes near the pyrenoid and 4 or more sublobes in the anterior region, not lobed posteriorly	2–3	[31]

**Table 2:** Comparison of SSU rDNA sequences between the isolated strains of *Tetraselmis jejuensis* and other *Tetraselmis* species, such as *T. carteriiformis*, *T. subcordiformis*, *T. chuii*, *T. suecica*, and *T. convolutae*. The numbers indicate different mismatched base pairs (bp) from each other. The numbers in parentheses indicate dissimilarity (%) including gaps. The bold numbers represent the highest similarity among them.

Accession Number	FJ559384	FJ559380	DQ207405	MK541745	U05039
Species	*T. carteriiformis*	*T. subcordiformis*	*T. chuii*	*T. suecica*	*T. convolutae*
*Tetraselmis* sp. DJ 2-1	22 (1.38)	21 (1.31)	18 (1.11)	**17 (1.05)**	29 (1.79)
*Tetraselmis* sp. DJ 2-4	23 (1.48)	22 (1.41)	20 (1.28)	**19 (1.22)**	31 (1.98)
*Tetraselmis* sp. DJ 2-5	24 (1.50)	23 (1.44)	20 (1.25)	**19 (1.19)**	31 (1.93)
*Tetraselmis* sp. DJ 2-2	29 (1.85)	28 (1.78)	25 (1.59)	**24 (1.53)**	36 (2.28)
*Tetraselmis* sp. YO 3-4	22 (1.43)	21 (1.36)	18 (1.17)	**17 (1.10)**	29 (1.87)
*Tetraselmis* sp. YO 3-3	25 (1.56)	24 (1.50)	21 (1.31)	**18 (1.13)**	32 (1.99)
*Tetraselmis* sp. YO 3-5	25 (1.53)	24 (1.47)	22 (1.32)	**17 (1.06)**	34 (2.03)
*Tetraselmis* sp. YO 3-2	25 (1.53)	24 (1.47)	21 (1.27)	**17 (1.05)**	33 (1.98)
*Tetraselmis* sp. YO 3-1	25 (1.53)	24 (1.47)	18 (1.07)	**17 (1.05)**	33 (1.96)
*Tetraselmis* sp. YO 4-1	22 (1.36)	21 (1.30)	18 (1.10)	**17 (1.04)**	29 (1.77)
*Tetraselmis* sp. YO 4-2	22 (1.36)	21 (1.30)	18 (1.11)	**17 (1.05)**	29 (1.79)
*Tetraselmis* sp. YO 5-5	24 (1.48)	23 (1.42)	23 (1.39)	**17 (1.06)**	35 (2.11)
*Tetraselmis* sp. YO 3-11	10 (0.98)	9 (0.88)	9 (0.88)	**6 (0.60)**	13 (1.27)
*Tetraselmis* sp. YO 3-12	26 (1.61)	25 (1.55)	22 (1.37)	**19 (1.19)**	33 (2.04)
*Tetraselmis* sp. YO 3-14	13 (1.22)	12 (1.13)	15 (1.39)	**11 (1.06)**	19 (1.76)
*Tetraselmis* sp. YO 2	3 (0.19)	**2 (0.13)**	21 (1.32)	20 (1.26)	28 (1.75)

**Table 3:** Collection details of *Tetraselmis jejuensis* isolated from two supralittoral tide pools, Daejeong (DJ) and Yongduam (YO), at Jeju Island in April and June 2019. The salinity and temperature measured at the time are indicated along with the date and location.

Strains	Sampling Date	Salinity (%)	Temperature (°C)	Location	Latitude	Longitude
*Tetraselmis* sp. DJ 2-1	11 Apr 2019	0.5	19.0	Daejeong	33.2126	126.2948
*Tetraselmis* sp. DJ 2-4		0.5	19.0	Daejeong	33.2126	126.2948
*Tetraselmis* sp. DJ 2-5		0.5	19.0	Daejeong	33.2126	126.2948
*Tetraselmis* sp. DJ 2-2		0.5	19.0	Daejeong	33.2126	126.2948
*Tetraselmis* sp. YO 3-4		0.3	20.8	Yongduam	33.5159	126.5120
*Tetraselmis* sp. YO 3-3		0.3	20.8	Yongduam	33.5159	126.5120
*Tetraselmis* sp. YO 3-5		0.3	20.8	Yongduam	33.5159	126.5120
*Tetraselmis* sp. YO 3-2		0.3	20.8	Yongduam	33.5159	126.5120
*Tetraselmis* sp. YO 3-1		0.3	20.8	Yongduam	33.5159	126.5120
*Tetraselmis* sp. YO 4-1	16 Jun 2019	0.8	29.9	Yongduam	33.5159	126.5120
*Tetraselmis* sp. YO 4-2		0.8	29.9	Yongduam	33.5159	126.5120
*Tetraselmis* sp. YO 5-5		1.6	30.4	Yongduam	33.5159	126.5120
*Tetraselmis* sp. YO 3-11		3.1	29.4	Yongduam	33.5159	126.5120
*Tetraselmis* sp. YO 3-12		3.1	29.4	Yongduam	33.5159	126.5120
*Tetraselmis* sp. YO 3-14		3.1	29.4	Yongduam	33.5159	126.5120
*Tetraselmis* sp. YO 2		3.9	29.5	Yongduam	33.5159	126.5120

**Table 4:** Universal eukaryotic primers used to amplify the SSU, ITS, and LSU regions of ribosomal DNA.

Primer Name	Primer Region	Sequence (5'–3')	Reference
EukA	Forward, SSU	AACCTGGTTGATCCTGCCAG	[55]
G18R	Reverse, SSU	GCATCACAGACCTGTTATTG	[56]
570F	Forward, SSU	GTAATTCCAGCTCCAATAGC	[57]
EukB	Reverse, SSU	TGATCCTTCTGCAGGTTCACCTAC	[55]
ITSF2	Forward, ITS	TACGTCCCTGCCCTTTGTAC	[56]
LSUB	Reverse, LSU	ACGAACGATTTGCACGTCAG	[56]
LSU500R	Reverse, LSU	CCCTCATGCTACTTGTTTGC	[56]
LSU500F	Foward, LSU	GCAAACAAGTACCATGAGGG	[56]
Euk1209F	Forward, ITS	GGGCATCACAGACCTG	[58]
ITSFR2	Reverse, ITS	TCCCTGTTCATTCGCCATTAC	[56]
1483R	Reverse, LSU	GCAAACAAGTACCATGAGGG	[59]

## Data Availability

The data presented in this study are available on request from the corresponding authors. In addition, the data that support the findings of this study are openly available in GenBank with the accession numbers MZ435987, MZ435988, MZ435989, MZ435990, MZ43591, MZ435992, MZ435993, MZ435994, MZ435995, MZ435996, MZ435997, MZ435998, MZ435999, MZ436000 and MZ436001.
